# Preparation of Cellulose/Laponite Composite Particles and Their Enhanced Electrorheological Responses

**DOI:** 10.3390/molecules26051482

**Published:** 2021-03-09

**Authors:** Zhao Liu, Zhenjie Zhao, Xiao Jin, Li-Min Wang, Ying Dan Liu

**Affiliations:** State Key Lab of Metastable Materials Science and Technology, and Collage of Materials Science and Engineering, Yanshan University, Qinhuangdao 066004, China; liuzhao0120@sina.cn (Z.L.); zhenjie_shine@163.com (Z.Z.); jinxiao94315@163.com (X.J.); limin_wang@ysu.edu.cn (L.-M.W.)

**Keywords:** cellulose, Laponite, composite, electrorheological fluid, sedimentation stability

## Abstract

Cellulose, as a natural polymer with an abundant source, has been widely used in many fields including the electric field responsive medium that we are interested in. In this work, cellulose micron particles were applied as an electrorheological (ER) material. Because of the low ER effect of the raw cellulose, a composite particle of cellulose and Laponite was prepared via a dissolution–regeneration process. Scanning electron microscopy (SEM), Fourier-transform infrared spectroscopy (FT-IR) and X-ray diffraction (XRD) were used to observe the morphologies and structures of the composite particles, which were different from pristine cellulose and Laponite, respectively. The ER performances of raw cellulose and the prepared composite were measured by an Anton Paar rotational rheometer. It was found that the ER properties of the composite were more superior to those of raw cellulose due to the flake-like shapes of the composite particles with rough surface. Moreover, the sedimentation stability of composite improves drastically, which means better suspension stability.

## 1. Introduction

Electrorheological (ER) fluids are a kind of smart complex fluid which can respond to an electric field and show electric-field-controllable viscosity [1,2]. They usually consist of dielectric particles and insulating oil. The significant increase in viscosity of ER fluids is aroused by polarization and alignment of the dielectric particles in the direction of the electric field, which also results in a state transition from fluid-like to solid-like upon the stimuli of the electric field. These electric-field-controllable properties of ER fluids give them application potential in the fields of shock absorbing [3], damping [4], braking [5], finishing [6] etc.

In ER fluids, silicone oil is normally applied as the carrier liquid due to its good stability and diverse accessible viscosities. While the range of dielectric particles applied in ER fluids are wide, covering many kinds of materials like inorganics [7,8], semi-conducting polymers [9], natural polymers [10] and polyelectrolytes [11], for real applications, the field-induced change in viscosity or shear stress, which is called ER effect, is the main evaluation criterium of ER fluids. Thus, high and stable ER effect is what researchers want to pursue in the study of ER Fluids. For this purpose, one of the strategies is to design hybrid or composite materials to improve the dielectric polarizability of the dispersed particles. In addition, easy accessibility or facile synthesis of dispersed particles is also necessary for the wide use of ER fluids. From this point of view, natural polymers are attractive candidates as ER materials because of their abundant source, low price, available chemical modification and biodegradability. To date, various natural polymers including cellulose [12,13,14], chitosan [15,16], starch [17,18], and algae [19,20] particles have been applied as ER materials. However, most of them exhibited relative low ER effects compared with some outstanding inorganic ER materials. To improve the ER effect of natural polymer-based ER materials, chemical modification is usually used. Choi et al. reported phosphate cellulose as an anhydrous ER material which showed high ER effect [21]. Ko et al. also applied acid and acid/urea complex groups to modify chitosan particles to improve their ER effect [22]. It is also reported that the ER effect of chitosan depends on the degree of deacetylation [23].

Comparing with chemical modification, introducing inorganic nanomaterials into polymer matrix (or particle blending) is a facile and effective way to improve the ER properties of polymers, which also brings other superiorities, for example enhanced thermal stability [24]. When semi-conducting polymer is used as the matrix, in situ polymerization [25] and Pickering emulsion polymerization [26,27] have been widely used in preparing inorganics blended composite ER materials. In addition to silica and titania nanoparticles, 2-dimentional nanosheets such as clay and graphene (or graphene oxide) are the mostly applied inorganics. For natural polymer-based ER materials, blending with inorganics is rarely used because of the solid nature and high molecular weight of the natural polymers. Hu et al. reported a new ER material of chitosan-decorated graphene nanosheets by microwave-assisted treatment [28]. It implies the possibility of preparing inorganics-blended natural polymer particles as ER material.

Cellulose is a polysaccharide with linear chain of D-glucose units and grafted —OH groups. Because of its abundance of origin and sustainability, accessibility of chemical modification, low cost and non-toxicity, cellulose has attracted much attention both in research and engineering applications. Polar groups of —OH in cellulose provide it with electro-responsive properties as well as accessibility of reaction. That is why cellulose has been applied as ER materials. In the recent development in the processing of cellulose, dissolution–regeneration has become a useful way to prepare cellulose/inorganics composite materials by introducing inorganic nanomaterials into the cellulose solution [29,30], which also gives us inspiration for the preparation of new cellulose-based ER materials. In this study, we introduce Laponite, a synthetic clay type with 2-dimentional disk shape (thickness: 1 nm; diameter: 25 nm), into cellulose particles by the dissolution–regeneration process of cellulose. In a previous study, Laponite as well as many other kinds of clays were used as ER materials separately or blended with polymers [31,32,33]. The special layered structures of clays played significant roles in preparing composite particles with intercalated, exfoliated, and even core–shell structures [34,35,36]. Herein, because of the existence of Laponite nanosheets, the arrangement of cellulose polymer chains in regeneration process is hindered, which resulted in different crystal structures in the composite. In addition, the dielectric properties of Laponite can also contribute to the ER effect of the composite particles.

## 2. Results and Discussions

**Figure 1:**
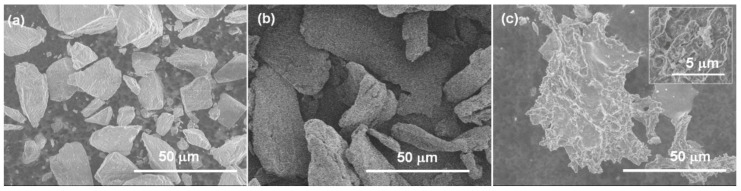
SEM images of Laponite (**a**), cellulose (**b**) and cellulose/Laponite composite (**c**). The inset of (**c**) is the cellulose/Laponite particles observed at a larger magnitude.

The morphologies of cellulose, Laponite and the cellulose/Laponite composite particles are shown in Figure 1. The Laponite particles (Figure 1a) are in an irregular shape with an average diameter of about 20 μm, which means the layered disks of Laponite are stacked to form micron particles in the dry powder form. The surface conditions of Laponite particles are very bumpy. Raw cellulose particles in Figure 1b are rod-like shape with diameters of about 10–30 micrometers and are 50–100 micrometers in length. The composite particles of cellulose/Laponite are formed via the regeneration of cellulose in alkali solution. Because of the fast precipitation and vigorous stirring, the regenerated cellulose particles easily form porous structures with rough surfaces which has been observed in previous study [37]. Herein, the composite particles of cellulose/Laponite shown in Figure 1c are flake-like with larger particle size than raw cellulose. In addition, compared with the relatively smooth surface of raw cellulose, those composites are extremely rough with many pores and nanoparticles on the surface.

**Figure 2:**
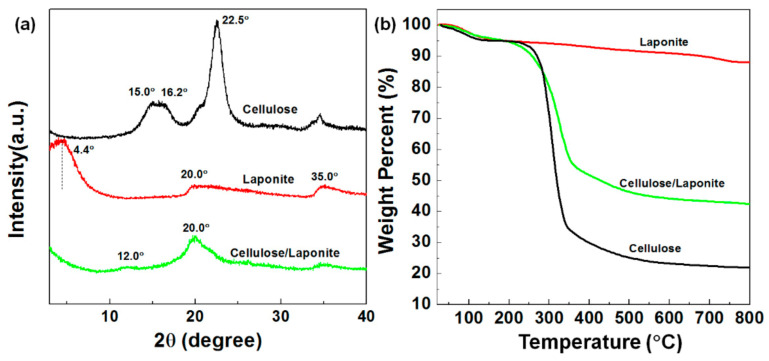
XRD patterns (**a**) and TGA curves (**b**) of cellulose, Laponite and cellulose/Laponite composite.

Figure 2a shows the XRD patterns of Laponite, cellulose and cellulose/Laponite composite. The characteristic diffraction peaks of cellulose are at 2θ = 15.0°, 16.2 and 22.5°, which are the symbolic peaks of cellulose I. The diffraction peaks of Laponite are located at 2θ = 4.4°, 20.0° and 35.0°, reflecting the crystal planes of (001), (100) and (110) [38,39,40]. According to the 2θ of (001), the basal spacing of Laponite is calculated to be 2 nm, which is much larger than the theoretical value of 0.96 nm. This may be due to the moisture that is absorbed in the interlayer of Laponite. After compounding, the main peaks of the composite are at 2θ = 12.1°, 19.9° and 21.8°, indicating that, after alkali treatment, cellulose I transforms into the form of cellulose II [37]. The sharp peak observed in the XRD pattern of Laponite at 2θ = 4.4° disappears, which means the cellulose chains are intercalated into the interlayers of clays. Other characteristic peaks of Laponite are observed in the XRD pattern of the composite, which confirms the successful blending of Laponite with cellulose. Another obvious characteristic of the composite is that there is an apparently sharp less peak at 35° compared with the peak that is shown by raw cellulose. Based on the Scherrer Formula, a sharper peak means a higher degree of crystallinity and better orientation. The reduced diffraction intensity is due to the introduction of Laponite in the matrix of cellulose, which hinders the re-generation of highly oriented crystalline areas of the cellulose matrix.

The thermogravimetric curves of Laponite, raw cellulose and their composite are shown in the Figure 2b. For the three samples, less than 5% weight loss is observed before 120 °C caused by the loss of absorbed water. As the temperature reaches 400 °C, large weight loss up to 50% and 70% appears in the curve of the cellulose/Laponite composite and raw cellulose, respectively, due to the degradation of the carbohydrate backbone. For Laponite, there is no obvious weight loss in this temperature range because of the high heat resistance of phyllosilicates. As the temperature increases to 800 °C, the residual mass for each sample is about 88% (Laponite), 43% (cellulose/Laponite) and 22% (cellulose). The higher residual mass of the composite than that of raw cellulose confirms that Laponite is introduced to the cellulose matrix. 

**Figure 3:**
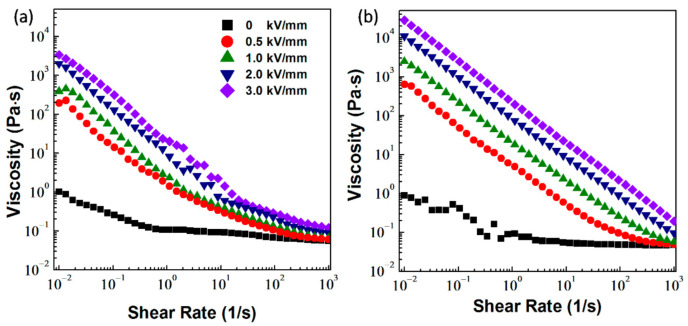
Shear viscosity curves of the electrorheological (ER) fluids of cellulose (**a**) and cellulose/Laponite composite (**b**) as a function of shear rate measured at different electric field strengths.

Figure 3 presents the shear viscosity of the ER fluids measured in the shear rate range of 0.01–1000 s^−1^. Before application of an electric field (E = 0 kV/mm), the solid cellulose or cellulose/Laponite particles randomly dispersed in silicone oil. Thus, both ER fluids are more like Newtonian fluid with a constant viscosity when shear rate exceeds 1 s^−1^. At higher shear rate, the viscosity of the ER fluid of cellulose/Laponite composite is lower than that of the cellulose ER fluid, which may be related to the flake-like morphology of the composite or the affinity between the composite and silicone oil. As the electric field is applied, the suspended particles are polarized and align in the direction of electric field driven by the electrostatic interaction between particles. The inner fibril structures result in sudden increase in shear viscosity of the ER fluids. When the rotor starts to shear, the particle chains or columns incline to the direction of shear flow gradually and are sheared into chain segments as the shear rate becomes higher. That is the reason both ER fluids show obvious shear thinning phenomena in shear viscosity curves. For each ER fluid, the value of shear viscosity is enhanced significantly by increasing electric field strength. When comparing the two ER fluids, it can be seen that, at an electric field strength, the viscosity curve of the composite ER fluid is much smoother and higher, indicating that the composite particles can form more robust chains under an electric field.

**Figure 4:**
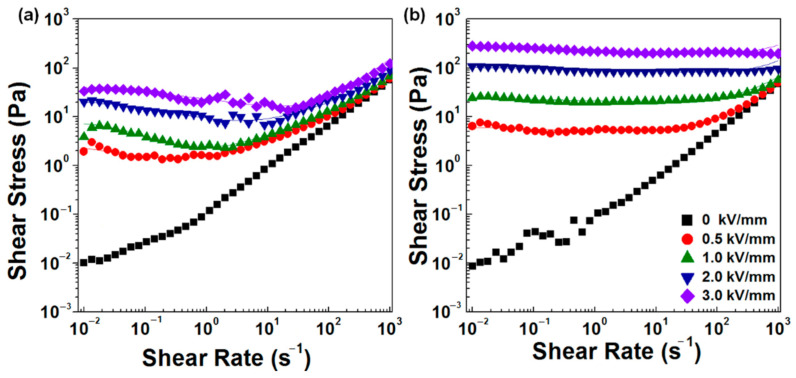
Shear stress curves of the ER fluids of cellulose (**a**) and cellulose/Laponite composite particles (**b**) at various electric field strengths. The solid lines are fitted from the CCJ model.

Figure 4 shows the shear stress of two ER fluids as a function of shear rate (0.01–1000 s^−1^). When the electric field is zero, the same as has been observed in viscosity curves, two ER fluids show the characteristics of Newtonian liquids in that the shear stress has a linear relationship with the shear rate. When an electric field is applied, the shear stress of cellulose ER fluid (Figure 4a) shows a plateau value in the low shear rate region (0.01–1 s^−1^) and then increase with the shear rate in the high shear rate region (1–1000 s^−1^). This is because low shear rate cannot destroy the particle chains totally due to the disrupt–rebuild competition process of particle chains. The increase in shear stress at high shear rate is attributed to the strong hydrodynamic force of shear flow which depends on shear rate and breaks the particle chains totally. For the ER fluid of the cellulose/Laponite composite (Figure 4b), the shear stress curves are independent of the shear rate. A steady plateau region over the whole shear rate range is observed for each shear stress curve. It means the electrostatic force between the composite particles dominates in the steady shear process with controlled shear rate. It is different from that which has been observed in the cellulose ER fluid. In addition, the shear stress values for the ER fluid of cellulose/Laponite are much higher than that of the cellulose ER fluid. 

To analyze the shear stress curves further, we use a constitutive equation to fit the shear stress curves of the two ER fluids. It is a six-parameter-equation named the Cho–Choi–Jhon (CCJ) model and has played a significant role in analyzing flow curves of ER fluids in previous studies, especially for the flow curves with typical shear rate dependent characteristics [41,42]. The equation of the CCJ model is written as follows:

(1) $$\tau =\frac{\tau_{y}}{1+{\left(t_{2}\dot{\gamma }\right)}^{\alpha }}+\eta_{\infty }(1+\frac{1}{{\left(t_{3}\dot{\gamma }\right)}^{\beta }})\dot{\gamma }$$

where *τ_y_* and *η_∞_* present the dynamic yield shear stress and viscosity at infinite shear rate, *t*_2_ and *t*_3_ are both time constants, *α* and *β* are the parameters to adjust the shape of the fitting curves. The fitting values of these important parameters are shown in Table 1. It can be seen that the CCJ model can fit the flow curves of the two ER fluids very well regardless of whether the curves depend on shear rate or not. 

**Figure 5:**
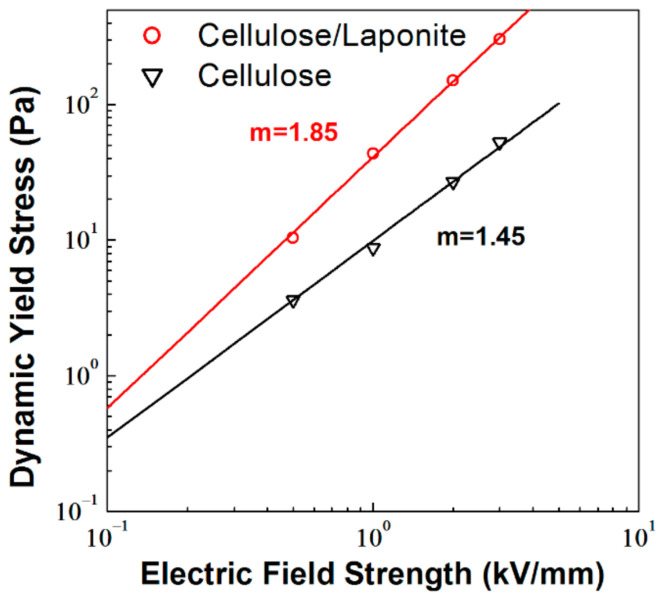
Dynamic yield stress (*τ_y_*) of the ER fluids as a function of electric field strength (*E*). The solid lines are fitted from the power law equation *τ_y_*∝ *E*^m^.

The dynamic yield stress (*τ_y_*) obtained from the fitting results is the crucial criterion to evaluate ER fluids. It greatly depends on the components of ER fluids and also exhibits close relation with electric field strength (*E*). As shown in Figure 5, the dynamic yield stress increases stepwise with *E*. It has been found to be a power law dependence, described as $\tau_{y}\propto E^{m}$ with a power exponent *m* [43,44]. The parameter *m* in this equation represents the ability of response to the electric field, the value of which ranges from 1.0 to 2.0 is mainly associated with the characteristics of the suspended particles. The dynamic yield stresses of the two ER fluids are replotted as a function of *E* in Figure 5. After fitting by the power law equation, the power exponent *m* is 1.45 for the raw cellulose ER fluid and 1.85 for the cellulose/Laponite composite ER fluid. It indicates that the composite has a higher field-dependent increase in dynamic yield stress.

Therefore, it is obvious that both yield stress and electric-field-dependence of the composite ER fluid are enhanced compared with the ER fluid of raw cellulose. It may be related to the morphological and chemical structures of the particles. It can be observed from Figure 1 that the composite particles have a rough surface which will enhance the interfacial polarizability of the particles. In addition, the intercalated Laponite nanosheets in cellulose also have a positive effect on electro-responsive properties of the composite particles because of their own ER properties [45,46], which has been confirmed in a previous study [36]. 

**Figure 6:**
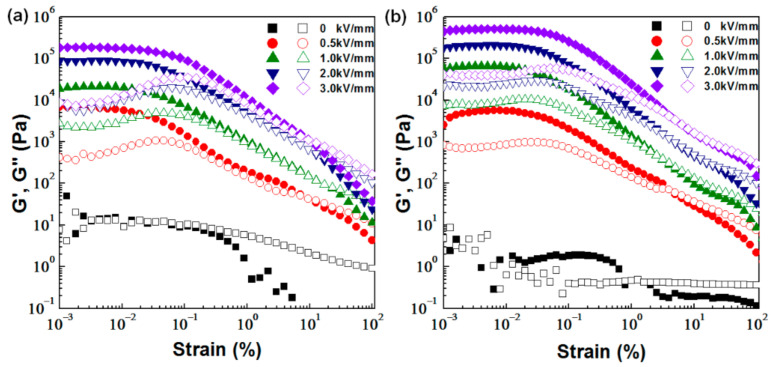
Amplitude sweeps of ER fluids based on cellulose (**a**) and cellulose/Laponite composite (**b**) measured at various electric field strengths (G’: closed symbol; G”: open symbol).

Figure 6 illustrates the rheological properties of the ER fluids of cellulose and cellulose/Laponite composite particles, measured in a dynamic amplitude sweep mode with a constant frequency of 10 rad/s. The storage modulus (G’) and loss modulus (G”) can be obtained in the amplitude oscillatory tests. According to the dependence of G’ and G” on amplitude, the linear viscoelastic (LVE) region of the ER fluids under the electric field are defined. It is the strain range where G’ is independent of strain because of the elastic deformation of the system. As shown in Figure 6a,b, when the electric fields are not applied, the values of G’ and G” are very low and almost equivalent before the strain of 0.1%. It means the ER fluid without the stimuli of the electric field is more like a viscoelastic liquid. As mentioned above, after applying an electric field, the particles in ER fluids form chains or columns [47,48]. That is why G’ increases by several orders of magnitude and remains constant until strain amplitude reaches a certain value, which is the LVE region. A similar LVE region up to 0.03% is detected for the two ER fluids. In addition, compared with G”, G’ becomes predominant before a critical strain, indicating that the system performs like viscoelastic solid because of the fibril structures formed by the particles. As the electric field is higher than 0.5 kV/mm, both G’ and G” of the cellulose/Laponite ER fluid are higher than that of the cellulose ER fluid, which also implies that more robust chain structures are constructed by the composite particles under the same electric field strength.

**Figure 7:**
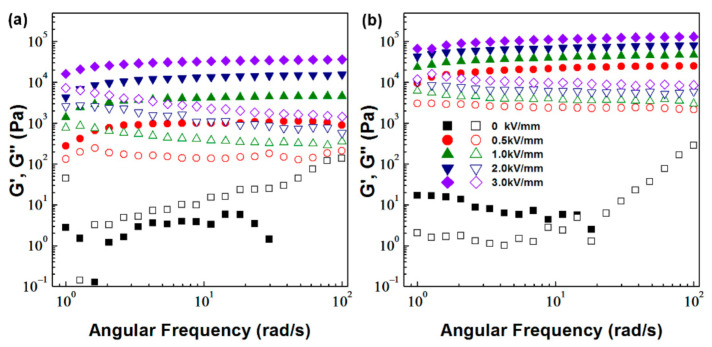
Frequency sweeps of the ER fluids based on cellulose (**a**) and cellulose/Laponite composite (**b**) measured at various electric field strengths (G’: closed symbol; G”: open symbol).

The results of the frequency sweep are illustrated in Figure 7. A constant strain of 0.003% in the LVE range is used to make sure that the structure of the viscoelastic solid is not destroyed in the oscillatory shear. The tests are conducted in the angular frequency range of 1–100 rad/s. On one hand, without electric fields, the G” increase with frequency and is higher than G’ in the whole frequency range (Figure 7a) or after a critical value (Figure 7b), showing the liquid characteristic of ER fluids. On the other hand, G’ and G” of the ER fluids are nearly constant at an applied electric field and G’ is much higher than G” over the entire frequency range, showing the viscoelastic solid properties. It is also observed that the G’ and G” of the cellulose/Laponite ER fluid are clearly higher than those of the raw cellulose ER fluid, indicating that stiffer chains or columns are formed in the cellulose/Laponite ER fluid. 

**Figure 8:**
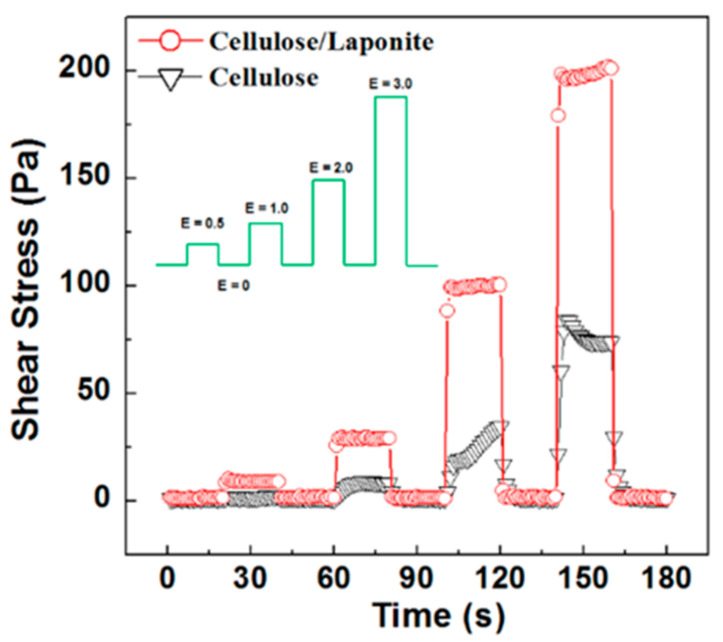
Shear stress curves of the ER fluids measured at a square-wave pulse electric field (shear rate: 1 s^−1^; time of each period: 20 s; unit of E: kV/mm).

The instant response of ER fluids can be gained through square-wave pulse voltage measurements. Figure 8 shows the shear stress of cellulose and cellulose/Laponite ER fluids at a shear rate of 1 s^−1^ and a square-wave pulse electric field (0.5–3 kV/mm) to observe the switching effect or sensitivity of the ER fluids to electric field. Because of the reversible liquid–solid transition in the ER fluid, the square-wave pulse in shear stress or viscosity can be observed according to the electric field in the same pattern. It can be found that the shear stress of the cellulose/Laponite ER fluid jumps up and down immediately at the switching point of the electric field, and shows a stable plateau value during each switch-on and switch-off period. For the raw cellulose ER fluid, climbing points are observed in the shear stress curve once the power supply is turned on, which is followed by lower and unstable shear stress. It confirms that the composite particles are more sensitive to the electric field than the raw cellulose particles.

**Figure 9:**
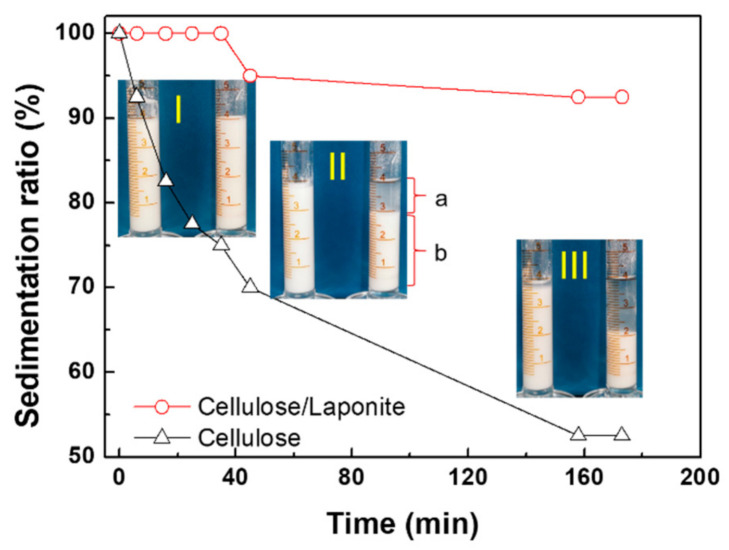
Sedimentation ratio of the two ER fluids as a function of time. Insets are the optical images of the ER fluids contained in glass tubes (left: cellulose ER fluid; right: cellulose/Laponite ER fluid).

Sedimental stability is a significant performance of ER fluids in practical application. Because of the density mismatch between the suspended particles and carrier liquid, the particles of ER fluids are apt to settle down towards the bottom of container. Herein, the density of the raw cellulose and cellulose/Laponite particles are 1.62 and 1.55 g/cm^3^, both of which are higher than that of silicone oil (density: 0.96 g/mL). However, the density of the composite particles is lower. As shown in Figure 9 (inset), both raw cellulose and the composite ER fluids are white liquids at the beginning. Then it is found that the cellulose particles settle down immediately in a few minutes and an upper supernatant part (a) is observed. The sedimentation ratio is defined as the percentage of the lower opaque part (b) to the total height of the liquid (a + b). For the composite ER fluid, an obvious supernatant layer is not observed until 40 min passes. While in this period, the sedimentation ratio of the raw cellulose ER fluid decreases rapidly and reaches 70%. Then in the next stage (40–160 min), the sedimentation ratio approaches nearly 50% for the raw cellulose ER fluid and is more than 90% for the cellulose/Laponite composite ER fluid. It is obviously that the sedimentation rate of the composite particles is much slower than that of raw cellulose particles, implying that the ER fluid of cellulose/Laponite has better suspension stability. One of the reasons for the improved anti-sedimentation property of the cellulose/Laponite particle is its lower density. The other is its flake-like shape and rough surface, both of which enhance the resistance suffered by the particle in the settling process. 

## 3. Materials and Methods

### 3.1. Materials

Cellulose (C6288, fibers, medium) were purchased from Sigma Aldrich. Laponite power (RD) applied in this work was bought from BYK, China. Silicone oil (viscosity: 50 cSt; density: 0.96 g/mL) (Beijing Hangping Guichuang Chemical Co., Ltd., Beijing, China), sodium hydroxide (NaOH, analytical purity) and alcohol (analytical purity) (Tianjin Kaitong Chemical Reagent Co. LTD, Tianjin, China) were employed directly without other treatments.

### 3.2. Preparation of Cellulose/Laponite Composite Particles

**Scheme 1:**
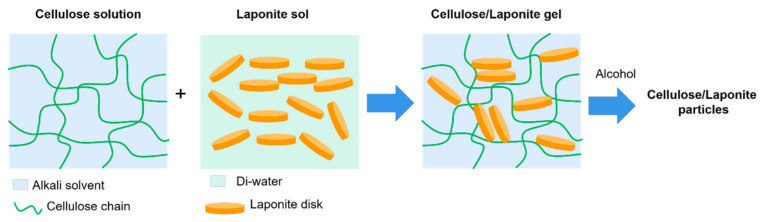
The preparation process for the cellulose/Laponite composite particles.

An amount of 1.0 g of Laponite was added into 100 mL water in batches at room temperature with the condition of intense stirring to form the stable sol system. An amount of 5.0 g of pristine cellulose was dispersed in an aqueous solution of NaOH (10 wt%) and precooled at the −10 ℃. After vigorous stirring, cellulose swelled in the alkali solution and formed a semitransparent and homogeneous solution. The Laponite hydrates were added into the cellulose system drop by drop at the stirring speed of 500 r/min. The composites of cellulose/Laponite were precipitated by adding alcohol and washed with deionized water several times. After freeze-drying, a white powder was obtained. A schematic process for preparing the cellulose/Laponite composite particles is shown in Scheme 1.

### 3.3. Characterization and Rheological Measurement

Morphologies of the raw cellulose, Laponite and the composite particles were observed by a scanning electron microscopy (SEM) (S4800, Hitachi, Tokyo, Japan). The crystal structures of the samples were characterized using a powder X-ray diffraction pattern (XRD) (MAX-2500PC, Rigaku, Tokyo, Japan) with a Cu-Kα radiation source. The thermal stability of the samples was determined using a thermogravimetric analyzer (TGA) (STA4993, Netzsch, Germany) with a heating rate of 10 °C/min in the temperature range of 25 to 800 °C in an air atmosphere.

Before rheological measurement, ER fluids of raw cellulose and cellulose/Laponite particles were prepared by dispersing the particles in silicone oil, respectively. Two uniform suspensions with the same mass fraction of 20% were obtained after ultrasonication. The rheological properties of the ER fluids were measured by a commercial rheometer MCR 502 (Anton Paar, Graz, Austria) with a concentric cylinder (CC) geometry and a DC power supply. The ER fluid was loaded between the gaps of the CC geometry. As the electric field was applied between the gaps, the field-induced change in rheological properties of the ER fluids could be sensed by the rotor of the rheometer. Steady shear flow and dynamic oscillation tests were used to observe the viscosity, shear stress and dynamic modulus of the ER fluids before and after the stimuli of an electric field.

## 4. Conclusions

We prepared cellulose/Laponite composite particles via a facile dissolution–regeneration method, during which process the Laponite clay was added in cellulose solution and restricted in the regenerated cellulose particles by adding a pore solvent. Compared with raw cellulose, the hybrid clay composite particles showed better ER effects, including higher shear stress, higher modulus, and faster response to electric fields. The enhanced ER effect of the cellulose/Laponite composite particles compared with raw cellulose is attributed to the inserted Laponite nanosheets into cellulose and the specific morphologies of the composite particles: flake-like shape with a rough surface. It was also found that the sedimentation stability of the composite ER fluid is significantly improved because of the surface morphology and the lower density of the particles. 

## Figures and Tables

**Table 1:** The fitting parameters of the Cho–Choi–Jhon (CCJ) model to the flow curves of raw cellulose ER fluid and cellulose/Laponite ER fluid.

Sample	Electric Field Strength (kV/mm)	*τ_y_*	α	β	t_2_	t_3_	η_∞_
Raw cellulose	0.5	3.60	0.55	0.067	65.16	0.0069	0.045
	1.0	8.76	0.81	0.64	18.84	0.0040	0.046
	2.0	26.88	0.46	0.61	9.87	0.0017	0.047
	3.0	52.72	0.30	0.20	2.59	0.0061	0.088
Cellulose/Laponite composite	0.5	10.41	0.092	0.57	2.63	0.107	0.042
	1.0	43.36	0.052	0.53	3.04	0.108	0.046
	2.0	150.10	0.085	0.80	0.0115	0.101	0.072
	3.0	302.30	0.150	0.33	0.0005	0.164	0.113

## Sample Availability

Samples of the compounds are not available from the authors.
